# Isolation and characterisation of lignin using natural deep eutectic solvents pretreated kenaf fibre biomass

**DOI:** 10.1038/s41598-024-59200-6

**Published:** 2024-04-15

**Authors:** Aatikah Meraj, M. Jawaid, Surendra Pratap Singh, Mohamed Mahmoud Nasef, Hidayah Ariffin, Hassan Fouad, Basim Abu‐Jdayil

**Affiliations:** 1 Laboratory of Bio-Polymer and its Derivatives, Institute of Tropical Forestry and Forest Products (INTROP), Universiti Putra Malaysia, 43400 UPM Serdang, Selangor Malaysia; 2 Chemical and Petroleum Engineering Department, College of Engineering, United Arab Emirates University (UAEU), P.O. Box. 15551, Al Ain, United Arab Emirates; 3 Grasim Industries Ltd, Kumarapatnam, Haveri, Karnataka 581123 India; 4 Department of Chemical and Environmental Engineering, Malaysia-Japan International Institute of Technology, Universiti Teknologi Malaysia, Jalan Sultan Yahya Petra, 54100 Kuala Lumpur, Malaysia; 5 Center of Hydrogen Energy, Institute of Future Energy, Universiti Teknologi, Jalan Sultan Yahya Petra, 54100 Kuala Lumpur, Malaysia; 6 Department of Bioprocess Technology, Faculty of Biotechnology and Biomolecular Sciences, Universiti Putra Malaysia, 43400 UPM Serdang, Selangor Malaysia; 7 Applied Medical Science Department, Community College, King Saud University, P.O Box 10219, Riyadh, 11433 Saudi Arabia

**Keywords:** Natural deep eutectic solvents, Lignin, Kenaf, Lignocellulosic biomass, Chemistry, Materials science

## Abstract

Extraction of lignin via green methods is a crucial step in promoting the bioconversion of lignocellulosic biomasses. In the present study, utilisation of natural deep eutectic solvent for the pretreatment of kenaf fibres biomass is performed. Furthermore, extracted lignin from natural deep eutectic solvent pretreated kenaf biomass was carried out and its comparative study with commercial lignin was studied. The extracted lignin was characterized and investigated through Infrared Fourier transform spectroscopy, X-ray Diffraction, thermogravimetric analysis, UV–Vis spectroscopy, and scanning electron microscopy. FTIR Spectra shows that all samples have almost same set of absorption bands with slight difference in frequencies. CHNS analysis of natural deep eutectic solvent pretreated kenaf fibre showed a slight increase in carbon % from 42.36 to 43.17% and an increase in nitrogen % from − 0.0939 to − 0.1377%. Morphological analysis of commercial lignin shows irregular/uneven surfaces whereas natural deep eutectic solvent extracted lignin shows smooth and wavy surface. EDX analysis indicated noticeable peaks for oxygen and carbon elements which are present in lignocellulosic biomass. Thermal properties showed that lignin is constant at higher temperatures due to more branching and production of extremely condensed aromatic structures. In UV–VIS spectroscopy, commercial lignin shows slightly broad peak between 300 and 400 nm due to presence of carbonyl bond whereas, natural deep eutectic solvent extracted lignin does not show up any peak in this range. XRD results showed that the crystallinity index percentage for kenaf and natural deep eutectic solvent treated kenaf was 70.33 and 69.5% respectively. Therefore, these innovative solvents will undoubtedly have significant impact on the development of clean, green, and sustainable products for biocatalysts, extraction, electrochemistry, adsorption applications.

## Introduction

Lignocellulosic biomass a naturally occurring, renewable, abundant source of polysaccharides and aromatic compounds. It comprises cellulose, hemicellulose, and lignin together form a complex and structured network. The complex network presents enormous potential to produce fuels and chemicals on a large scale, with significant economic benefits. Moreover, widespread availability and affordability of lignocellulosic biomass make it a promising and accessible resource for sustainable production processes across the world^1^. Lignocellulosic biomass is a chunk of an immense group of floras along with agricultural wastes like stalks and cobs of corn, bagasse of sugarcane and forest remains including sawdust, planer shavings, sander dust, bark, and chips in addition to waste left from woody products, and solid wastes materials like wastepaper and yard trashes^2,3^.

Even though these biomass reserves offer a sizable store of useful materials that are essential for developing biomass-based industry, more energy harvests are needed to keep up with the growing demand for bio-based fuel and chemicals. However, the challenge ahead with such biomass resources despite their low cost is converting them into bio-based chemicals by low-cost method then the fossil reserves progressions.

The lignocellulosic biomass available in sustainable and renewable feedstock can replace fossil fuels, chemicals, and other fossil-derived materials. Lignocellulose is an elementary structural component of all floras either woody or non-woody; most of them contain three main segments known as cellulose, lignin, and hemicellulose.

Currently, processing of lignocellulosic biomass usually includes use of common organic solvents, which are frequently utilized in chemical labs as well as in industries that raise serious concerns about safety, health, and environmental hazard^4,5^. Over the last decade, a great escalation has been seen in the global search for alternative solvents that could replace the traditional, harsh organic solvents with newer, less expensive, and greener agents^6–8^. Natural deep eutectic solvents (NADES) has emerged in the form of a green substitute.

The treatment with NADES of lignocellulose biomass rules out several hitches linked with traditional ionic liquids (ILs) like cost impact, energy-intensive recycling, and ecological concerns along with toxicity, sustainability, biocompatibility, and biodegradability. NADES are composed of organic acids, sugar alcohols, amines, amino acids, and other naturally occurring metabolites. Due to this, NADES is inherently superior to other conventional solvents such as ILs which are considered organic complexes that exist in liquid form at room temperature with lower melting points and are known as ionic liquids. NADES are widely available and are promising solvents that can be formed from natural-based chemicals. They are economical, simple to produce with high purity, recyclable, easy to handle, environment-friendly, and have low toxicity. Despite having such qualities that are quite identical to ILs, NADES has an advantage over this by being extremely selective and environmentally friendly. However, there is only one drawback of NADES which is a higher viscosity, which is one of the most significant properties and main obstacles to the application of NADES^6,9^. Furthermore, NADES has received a lot of attention and is quite popular in commercial applications, along with chemical industries, food, and pharmaceutical industries as well as enzyme and biofuel industries. Therefore, NADES is regarded as the solvent for the twenty-first century due to their potential applicability in a variety of industries^10,11^.

Particularly, kenaf (*Hibiscus cannabinus*) is one of the most industrial crops that are suitable for farming in humid weather conditions and is categorized under the family name Malvaceae. It is of indigenous origin in east-central Africa as a widespread wild plant throughout equatorial and tropical Asia and Africa^12^. Kenaf is generally introduced as an excellent producer of primitive ingredient fibre for paper, pulp as well as different fibre products^12^. It is utilized as a substitute for man-made fibres due to its numerous benefits such as natural occurrence, low cost, recyclability, and biodegradability^13^.

NADES are strongly related to the concepts of green chemistry and have received much more recent attention than other solvents. Therefore, NADES has been developed as a promising solvent system for the effective disintegration of lignocellulosic biomass and the separation of lignin. Researchers have recently become interested in NADESs because of their different combinations of two or three compounds mixed in different molar ratios. They could be used in a range of unexplored fields, where their unique properties might make them a potentially sustainable choice as functional solvents and matrices for end products.

Capitalizing on the valorisation of lignocellulosic biomass is favoured in an integrated biorefinery strategy, it is highly recommended for the separation and reclamation of significant products, it is the utmost valuable and energetic compounds that can be produced by lignocellulosic biomass constituents is lignin. In addition to being an excellent fuel source and having a high energy content, lignin is a great source of fuel and can be utilized in other high-value applications^14^.

Around, hundreds of various constituent-derived NADES reagents were created for the future usage of NADES mixture for numerous unidentified and unexplored fields of possible applications. Recently, widespread emphasis has been focused on the expansion and implementation of green biomass having renewable characteristics in a variety of manufacturing because of ongoing depletion in fossil fuels and the severity of ecological problems like global warming, etc. Although there are over 170 metric billion tonnes of biomass produced annually, from which a small portion is used as a chemical feedstock and is valued. To advance the biological adaptation of renewable feedstocks in terms of fuels and chemicals, significant efforts should be done such as starch, cellulose^15–17^, and lignin^18^.

Lignin is stable which connects cellulose and hemicellulose, and is the second-most common phenolic organic bio-based macromolecule after cellulose, which establishes an important composite in the cell wall of vascular plants and lignocellulosic resources^19^, and is available in large amounts in plants and is insoluble in water. It is produced annually in quantities of 10 metric billion tonnes and contributes about one-third of bio-organic carbon in the atmosphere^20^. Furthermore, lignin remains a significant outcome material of pulp and paper manufacturing^21^. The complex, heterogeneous, and amorphous polyphenolic typical structure of lignin, create the challenges in deployment of lignocellulose and consequently, it faces significant difficulties when broke down into higher-value products. Therefore, precise fractionation and pre-treatment approaches are needed to minimise the recalcitrance^22,23^. For the moment, plenty of functional groups are found in the structure of lignin, indicating possibilities of alteration to create useful intermediates and products^24^.

Tan et al. established research to examine the influence of multiple functional groups of acid hydrogen bond donor (HBD) of choline-chloride-derivated DES on lignin separation and obtained a greater lignin extraction yield^25^. The same type of study was done by Layman et al., observed that lignin was more soluble in DES-attached formic acid compared to acetic acid or Lactic acid^26^. However, various studies have increased interest in the implementation of DES as an optional solvent for lignin segregation, separation, alteration, and depolymerisation^27–30^. Furthermore, lignin isolated by DES implementation has also been shown to extract lignin with great yields, good purity, and lesser molecular mass, which are advantageous for future valorisation^27^. Also, DES-derivative extraction of lignin by splitting ether bonds has shown significant benefits in comparison to conventional ILs treatments, like as economical, eco-friendly, non-toxicity, etc.^29,31^.

To date, no research has been conducted so far on NADES pre-treated kenaf fibre from which lignin was extracted and was compared with commercial lignin. In this current work, we have established a NADES pre-treatment technique for kenaf fibres biomass that is over sufficient lignocellulosic crop for biomass deposits, specially cultivated in Asian terrain^32^. Kenaf fibre was treated with NADES solution using choline chloride (ChCl) as HBA and Lactic acid (LA) as HBD, and lignin was isolated from the pre-treated kenaf fibre. Further, the isolated lignin was characterised by to study of chemical identity, morphological changes, and thermal behaviours as well as its elemental analysis and XRD. Lastly, comparative study is carried out between the isolated lignin and commercial lignin.

## Materials and method

**Figure 1:**
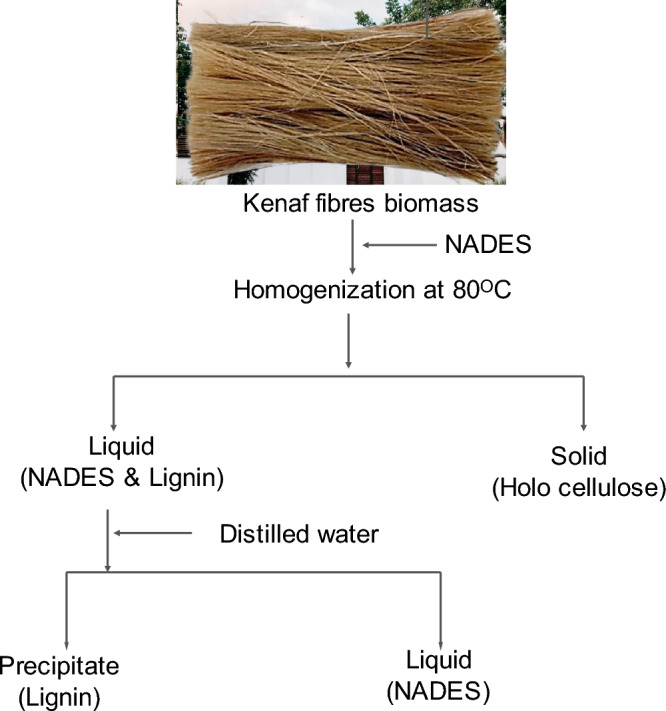
Schematic diagram of the lignin extraction process.

Kenaf fibres biomass was obtained from the Chuping plantation site of Kenaf venture global, Perlis Malaysia. Commercial lignin (Sigma-Aldrich), Choline chloride 98% (ChCl), Lactic acid (LA) ≥ 88%, Distilled Water (DW), and 95–98% H_2_SO_4_ (Sulphuric acid) were used for the pretreatment of kenaf fibre and extraction of lignin (Fig. 1). All these chemicals were purchased from Evergreen Sdn. Bhd., Malaysia.

### Lignocellulosic biomass preparation

Firstly, the kenaf biomass was prewashed with distilled water to eliminate any impurities and was kept overnight in an oven till the moisture content became lesser than 5% w/w. The dehydrated biomass was then hand cut into a small size up to 2–10 mm. The desired-sized biomass was utilised without any additional treatment.

### NADES preparation

The mixture of NADES solution was prepared using lactic acid/choline chloride (LA/CC) mixture. This mixture was prepared at a 2:1 molar ratio, and was kept on a constant shaking until a clear and translucent fluid solution was made within 10–15 min as explained by Día et al. and Francisco et al.^6,33^. Later, the kenaf fibre biomass was treated with NADES reagent for the extraction of lignin as described in the next section.

### Pretreatment of kenaf fibre

The pretreatment experiments were executed individually using biomass: NADES ratios of 1:12 and 1:15 molar ratios. The respective concentration of NADES was mixed with the biomass in a conical flask and the biomass was kept for digestion, with a total reaction volume maintaining a range (80–110 °C) of temperature. After digestion, the mixture was cooled down to 25 °C (room temperature), and the obtained NADES pretreated biomass dregs were vacuum-filtered using G_3_ Crucible.

### Extraction of lignin

The NADES pretreated Kenaf fibres were introduced to acid hydrolysis with 72% H_2_SO_4_ solution for 2 h at room temperature to hydrolyse and solubilize carbohydrates. The sample was subsequently diluted with distilled water to reduce the sulphuric acid concentration and was further kept for boiling for 4 h while continuing a fixed volume by the repeated addition of hot water. Following, the solution was kept overnight to settle down before being filtered and the residue was washed with hot water until its pH reaches neutral. Lastly, the insoluble residue (precipitate) was kept for drying until a constant weight was obtained which is the lignin content as discussed in TAPPI T222^34^, which is the most used analytical approach for determining the amount of lignin in lignocellulosic biomass.

### Ethical approval

No ethical clearance is required.

### Human and animal rights

We declare that there are no animal studies or human participant involvement in the study.

## Characterization of lignin

### Fourier transform infrared spectroscopy (FTIR)

FTIR spectra of raw, NADES pretreated kenaf fibre and NADES lignin extracted which was compared by commercial lignin; were achieved by using the KBr pellet method. A total of 32 scans per sample at a spectral resolution of 4 cm^−1^ were measured with a specific range (500–4000 cm^−1^).

### UV–VIS spectroscopy of NADES extracted lignin

NADES extracted lignin and commercial lignin were directly measured as solid form by clamping the sample with a UV–Visible spectrophotometer (UV-3600 series, Shimadzu) with an absorbance range of 220–800 nm with 1 nm spectra resolution.

### Scanning electron microscopy (SEM)

SEM images of untreated, NADES pretreated kenaf fibre and the NADES extracted lignin and commercial lignin were utilised to visualize and recognize the potential structural changes generated by pretreatment, using NOVA NANOSEM 230, FEI, Scanning Electron Microscope (SEM) equipped with EDX. Before viewing the samples, it was first stained with 2% uranyl acetate solution then mounted on the stub, coated with gold using a sputter coater, and observed with Scanning Electron Microscope.

### X-ray diffraction (XRD)

The XRD examination of untreated and NADES pretreated kenaf fibre biomass was done by using Cu radiation at 25 °C which was created at 30 kV and 30 mA. The diffraction spectra were obtained using the θ–2θ technique. The samples were continuously scanned at a specific angular range (20–70°), with a speed of 2 degrees/min at present of 0.6 s.

The crystallinity index (CrI) was calculated using Eq. 1 as described in^35^.

1 $${\text{The}}\;{\text{crystallinity}}\;{\text{index }}\left( \% \right) = \left( {Icry - Iamp} \right)/Icry*100$$

where *I* is the intensity of crystalline and amorphous regions.

### Thermogravimetric analysis (TGA)

Thermogravimetric study of NADES extracted lignin and commercial lignin was executed using TA instruments, TGA (Q500 V20. 13 Build 39) under an inert atmosphere using Al pans, temperature ranging from 25 to 700 °C at a heating rate of 10 °C min^-1^.

### Differential scanning calorimetry (DSC)

DSC curve was recorded using TA instruments DSC (Q20 V24.11 Build 124) between 0 and 300 °C with 10 °C min^−1^ heating rate.

## Results and discussion

### Chemical composition of Kenaf fibre and appearance of lignin

Table 1 shows chemical composition (wt%) of raw kenaf fibre. Generally, the cellulose concentration was higher in comparison to other compositions. The chemical composition of natural fibres is completely dependent on numerous characteristics such as plant origin and growing environments. Analysis of the chemical composition of raw kenaf fibre exhibited that extraction; cellulose, hemicellulose, and lignin were 1.20, 66.48, 19.41, and 6.79% respectively. In this study, lignin was isolated from pretreated kenaf fibre that had initially undergone NADES (LA/CC) treatment. The physical appearance of separated lignin was black. This appearance may be different for every isolation step and its distinguishing feature also may change.

**Table 1:** Chemical composition of kenaf fibre.

Sample	Extraction (wt%)	Cellulose (wt%)	Hemicellulose	Lignin (wt%)
Raw kenaf	1.20	66.48	19.41	6.79

### Fourier transform infrared spectroscopy analysis

Fourier transforms infrared (FTIR) spectroscopy of untreated (raw) and NADES pretreated kenaf fibre biomass, NADES extracted lignin, and commercial lignin was studied by direct transmittance using KBr pellet technique. FTIR analysis was examined to see the changes caused in NADES pretreated kenaf fibre biomass, also it is a reliable method for determining changes from functional groups and structural units of lignin composites^36^. It has evolved into an influential tool for determining the structure and offers a new explanatory and experimental framework for the study of complex natural polymer systems^37^. It quantified the presence of aromatic behaviour, C=O groups, and substitution patterns in the benzene ring^38^.

**Figure 2:**
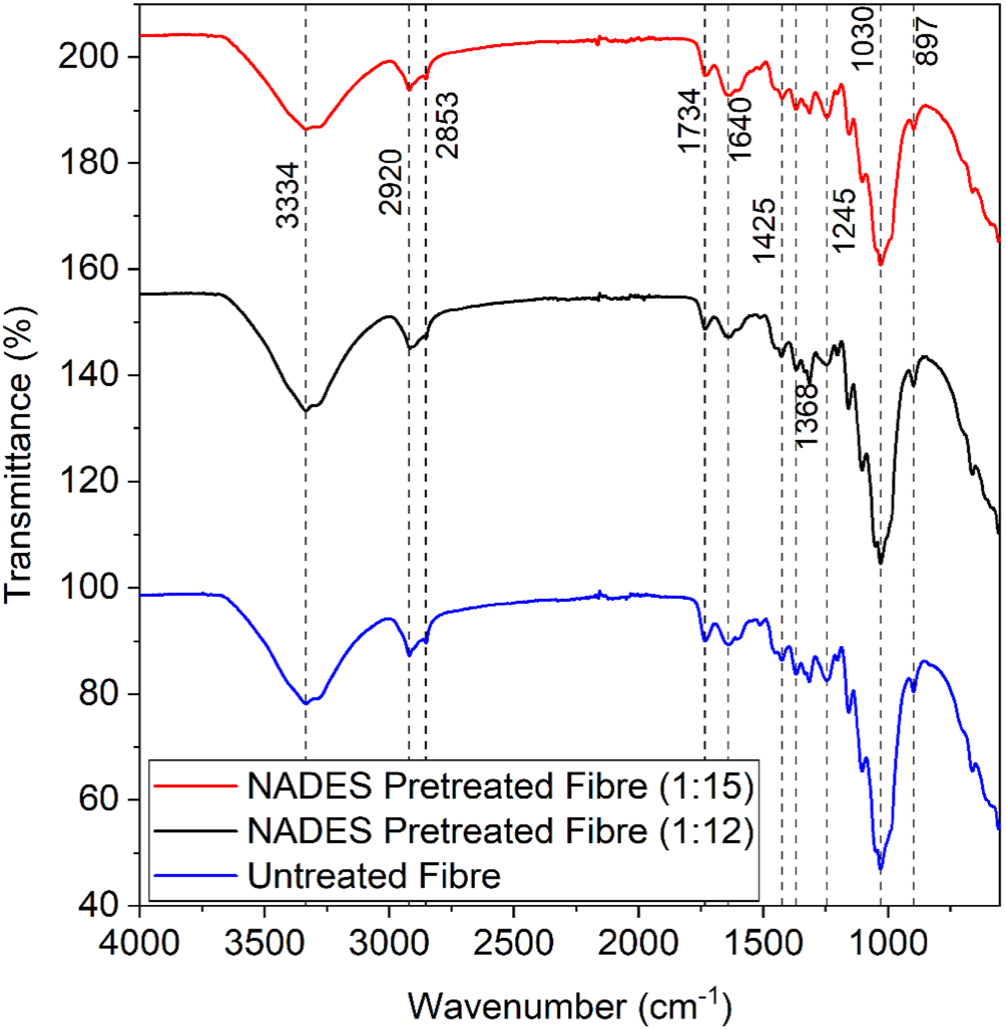
FTIR analysis of untreated and NADES pretreated kenaf fibres.

FTIR study of raw and NADES pretreated biomass was analysed and was done to examine the changes occurred due to NADES solution, and there were no major structural variations observed in the functional groups of biomasses as shown in Fig. 2. In general, the IR spectra of native or NADES pretreated fibres are measured in the range of 3000–3500 cm^−1^. For the kenaf fibre implemented in this study, the peak was found at 3332–3334 cm^−1^ for both untreated and NADES pretreated fibre (1:12 and 1:15 molar ratios) which is designated for the hydroxyl group stretching. The axial twist of the C-H group is associated with the observed absorption band at 2919, 2920, and 2917 cm^−1^ for untreated and NADES pretreated fibre (1:12 and 1:15 molar ratios).

The peak at around 1700 cm^−1^ attributes to carbonyl band (C=O) of hemicellulose in the kenaf fibre (untreated) and NADES pretreated fibre. The band at 1425 cm^−1^ is characteristic of the symmetric deformation of the CH_2_ group of cellulose, whereas the band at 1245 cm^−1^ denotes the C–O–C bond in the cellulose structure. The strong absorption band of C–OH at 1029–30 cm^−1^ was due to stretching vibration. The sharpness is often broader in lignin because of the –OH groups present in phenol and generally found inner scale of cellulose due to intermolecular hydrogen bonds. A slight variation in the peak can be seen because the different NADES and biomass ratios (1:12 and 1:15) were used and there were no major functional group changes among the spectra of raw and NADES pretreated biomass.

**Figure 3:**
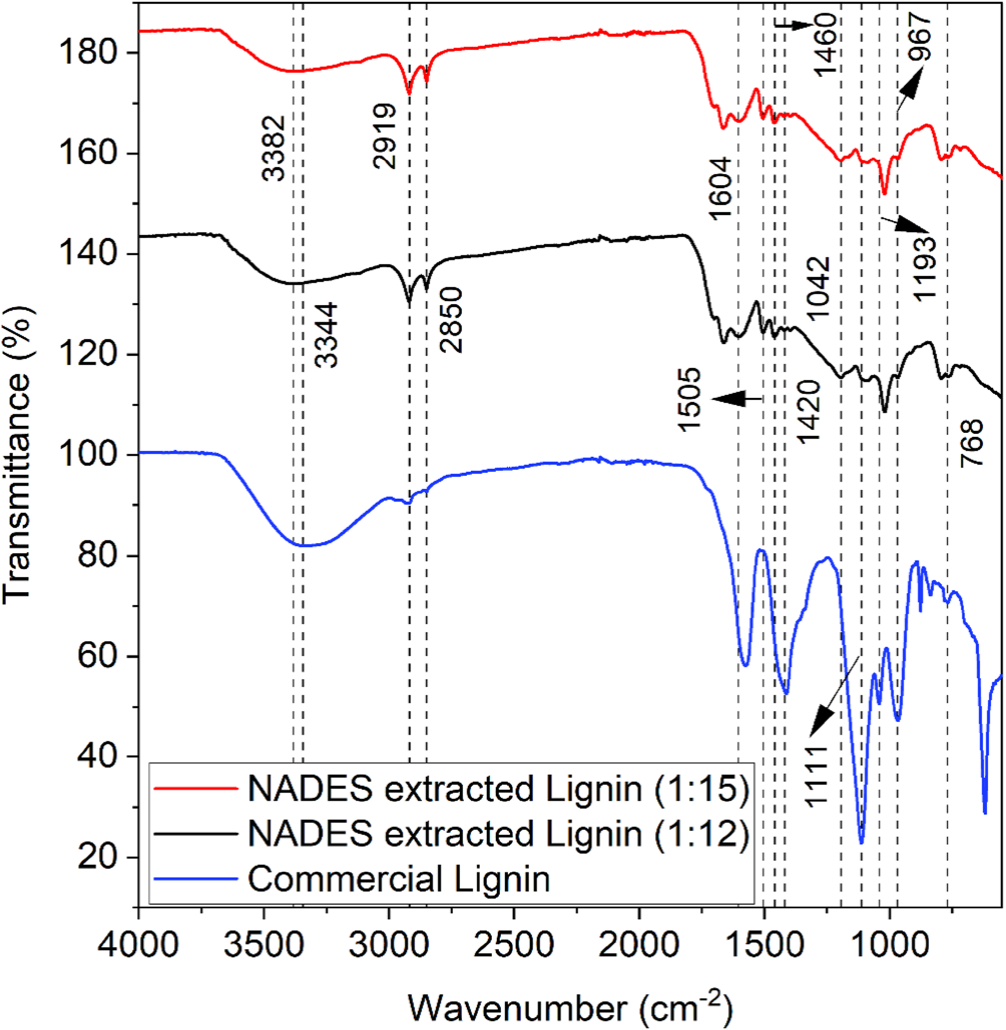
FTIR analysis of commercial lignin and NADES extracted lignin.

Figure 3 shows FTIR analysis of NADES extracted lignin of 1:12 and 1:15 molar ratios compared to commercial lignin. FTIR spectra shows all samples have almost same set of absorption bands with a slight difference in frequencies, though the intensities of some bands vary noticeably between samples. Other characteristics peaks were observed between 700 and 1700 cm^−1^, maximum absorption bands i.e., at 1604, 1505, 1460, 1420, 1193, 1111, 1042, 967, and 768 cm^−1^.

FTIR spectra of samples were examined, the presence of broad band ranging between 3500 and 3000 cm^−1^, relates to stretching vibrations of –OH^39^. Considering the maximum position of a particular band and its asymmetry, infers an addition to aliphatic OH groups, NADES extracted lignin as well as commercial lignin also contains a sizable amount of phenol-type hydroxyls. It was also observed that NADES extracted lignin displays a slight decrease in the –OH group stretching, the peak is small and wider as compared to commercial lignin. Absorption peak observed in both NADES extracted lignin at 2919 and 2850 cm^−1^ which is assigned to C–H stretching vibrations of CH_3_ and methylene groups, and a very small peak was observed in commercial lignin at around 2927 cm^−1^ which is related to CH_3_ and CH_2_ groups. The existence of spectral bands observed with an absorption maximum at 1460 and 1420 cm^−1^ produced by bending vibrations of CH_3_ and scissor vibrations of CH_2_ groups. The bands at 1604 and 1505 cm^−1^ which are defined as C=C, C=O stretching or bending vibrations of other groups were existing in NADES extracted lignin. The varying appearances in the spectra are due to different NADES and biomass ratios. The absorption bands at 500–1500 cm^−1^ are greater in NADES extracted lignin in comparison with commercial lignin.

### Elemental analysis

The CHNS analysis was performed on raw kenaf fibre and NADES pretreated kenaf fibre, summarised in Table 2, showed a slight increase in C (%) from 42.36 to 43.17%, and an increase in N (%) from − 0.0939 to − 0.1377% after NADES pretreated kenaf fibre. However, H and S (%) does not show any change.

**Table 2:** CHNS analysis of raw Kenaf fibre, NADES pretreated Kenaf fibre, NADES extracted lignin, and Commercial Lignin.

Name of samples	C%	N%	H%	S%
KF-RAW	42.36	− 0.0939	6.540	0.394
*KF-T	43.17	− 0.1377	6.687	0.380
CL*	17.35	0.3245	2.554	3.95
KF-L*	60.92	0.3092	5.831	0.545

*CL commercial lignin, KF-*L kenaf fibre NADES extracted lignin, *KF kenaf fibre.

Furthermore, CHNS analysis was also analysed of NADES extracted lignin compared with commercial lignin as shown in Table 2; the total percentage of carbon, nitrogen, hydrogen, and sulphur. A sharp variation of C (%) in NADES extracted lignin as compared to commercial lignin was observed. This could be due to the pretreatment of fibre with NADES before extraction of lignin. On the other hand, S (%) in NADES extracted lignin followed an opposite trend i.e., it decreased as compared to commercial lignin. However, no change was observed in N (%).

### Scanning electron microscopy (SEM)

**Figure 4:**
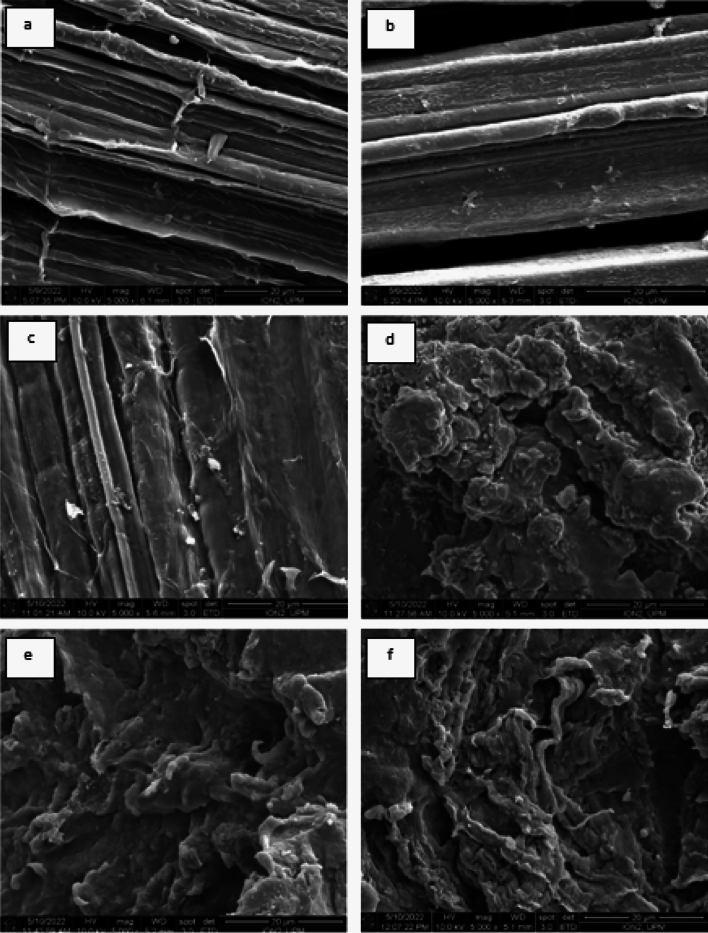
(**a**–**f**) demonstrate SEM images of (**a**) untreated kenaf fibre, (**b** and **c**) NADESs pretreated kenaf fibre (1:12 and 1:15 molar ratio), (**d**) commercial lignin, and (**e** and **f**) NADES extracted lignin obtained from 1:12 and 1:15 molar ratio.

In Fig. 4a displays SEM images of unprocessed or raw kenaf fibre, in which the bundles of kenaf fibre seen bonded together by lignin, and the surface of raw kenaf fibre appears rough and compact. In Fig. 4b–c after the fibre was treated with NADES solution (1:12 and 1:15 molar ratios respectively) the surface and texture of fibre appear smooth. However, some regions of Fig. 4c shows that surface of NADES pretreated kenaf fibre becomes a little bit rougher, which could be due to devolatilization/decomposition of the organic component of kenaf fibre, without affecting the lignin present on fibre, even after the pretreatment of fibre with NADES solution.

However, we have also compared SEM analysis of NADES extracted lignin obtained from NADES pretreated kenaf fibre with both the molar ratios i.e., 1:12 and 1:15 with commercial lignin which is shown in Fig. 4d–f above. It can be observed that commercial lignin shows irregular/uneven surfaces while, after the treatment of fibre with NADES solution, the NADES extracted lignin shows a smooth and wavy surface. The SEM structure Fig. 4d–f of commercial lignin differed significantly from the structure of isolated lignin from NADES pretreated kenaf fibre, this difference can be due to the treatment of raw kenaf fibre by NADES (LA: CC) solution and the NADES lignin extracted from it. SEM images of untreated and NADES pretreated kenaf fibres together with commercial and NADES extracted lignin as shown in Fig. 4a–f.

**Figure 5:**
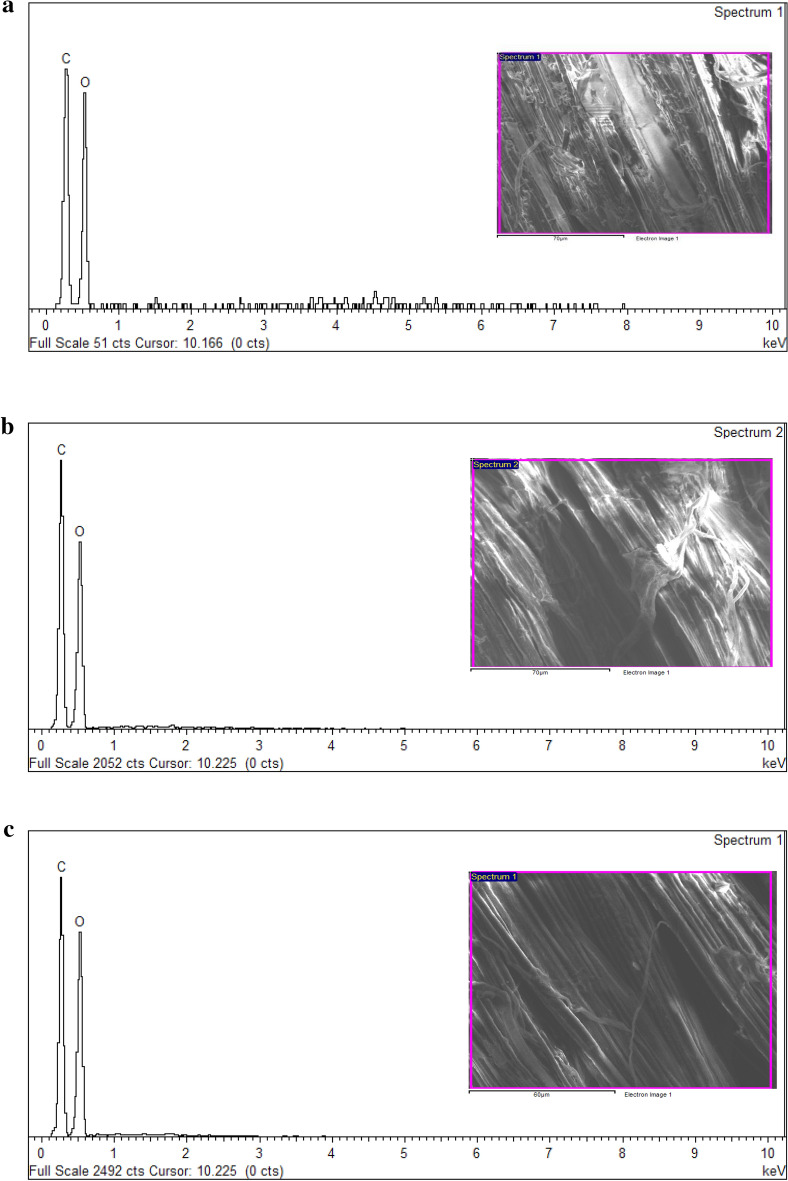

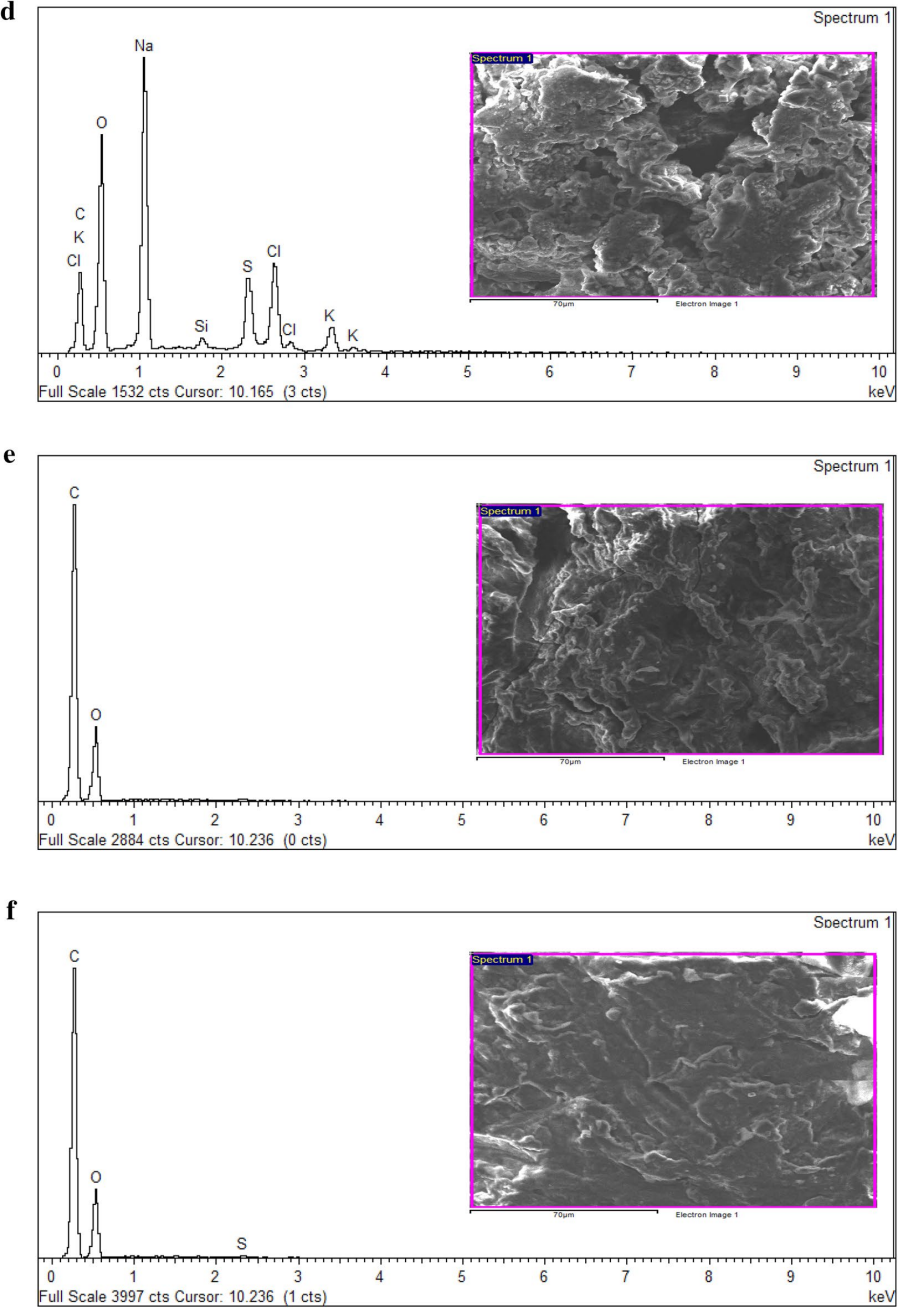
(**a**) EDX spectra of untreated (raw) kenaf fibre. (**b**) EDX spectra of NADESs pretreated kenaf fibre obtained from 1:12 molar ratio. (**c**) EDX spectra of NADESs pretreated kenaf fibre obtained from 1:15 molar ratio. (**d**) EDX spectra of commercial lignin. (**e**) EDX spectra of NADES extracted lignin obtained from 1:12 molar ratio. (**f**) EDX spectra of NADES extracted lignin obtained from 1:15 molar ratio.

In EDX analysis of raw and pretreated kenaf fibre are presented in Fig. 5a–c respectively, and their numerical data are summarised in Table 3 which indicates noticeable peaks for oxygen and carbon elements, representative of the standard structure of lignocellulosic biomass. This analysis was also studied in commercial lignin and NADES extracted lignin obtained from NADES pretreated kenaf fibre (1:12 and 1:15 molar ratios) shown in Fig. 5d–f respectively, and elemental composition is summarised in Table 4. Minute traces of element Si were only found in NADES pretreated kenaf of 1:15 molar ratio whereas, different elements were only seen in commercial lignin.

**Table 3:** Elemental composition of kenaf fibre.

Element	Raw kenaf fibre		NADES pretreated kenaf fibre (1:12)		NADES pretreated kenaf fibre (1:15)	
	Wt. (%)	Atomic (%)	Wt. (%)	Atomic (%)	Wt. (%)	Atomic (%)
C	44.53	51.68	48.42	55.56	46.28	53.44
O	55.47	48.32	51.58	44.44	53.72	46.56

**Table 4:** Elemental composition of commercial lignin and NADES extracted lignin.

Element	Commercial lignin		NADES extracted Lignin (1:12)		NADES extracted Lignin (1:15)	
	Weight (%)	Atomic (%)	Weight (%)	Atomic (%)	Weight (%)	Atomic (%)
C	24.12	34.67	64.11	70.41	64.29	70.66
O	38.95	42.04	35.89	29.59	35.43	29.23
Na	19.23	14.44	–	–	–	–
Si	0.59	0.36	–	–	0.28	0.12
S	5.66	3.05	–	–	–	–
Cl	8.40	4.09	–	–	–	–
K	3.04	1.34	–	–	–	–

### Thermal analysis

**Figure 6:**
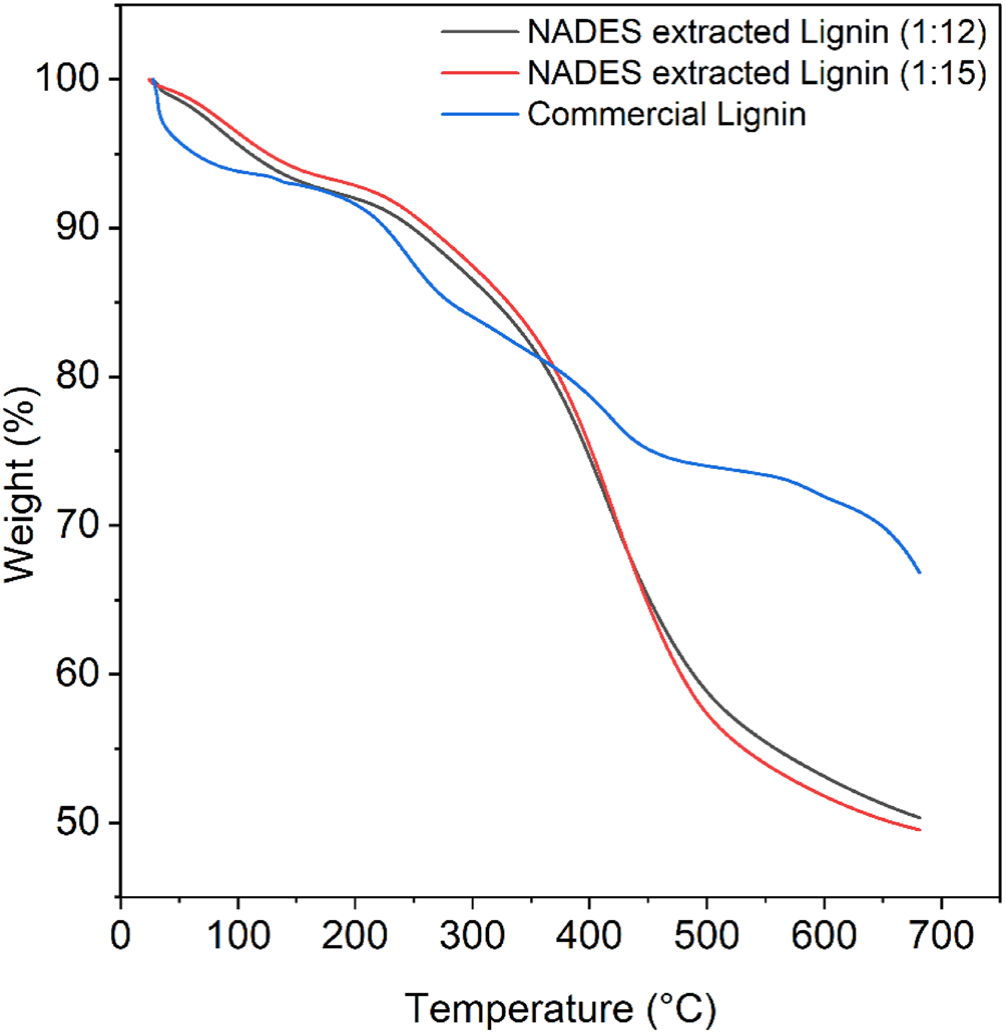
Thermograms of commercial lignin and NADES extracted lignin.

**Figure 7:**
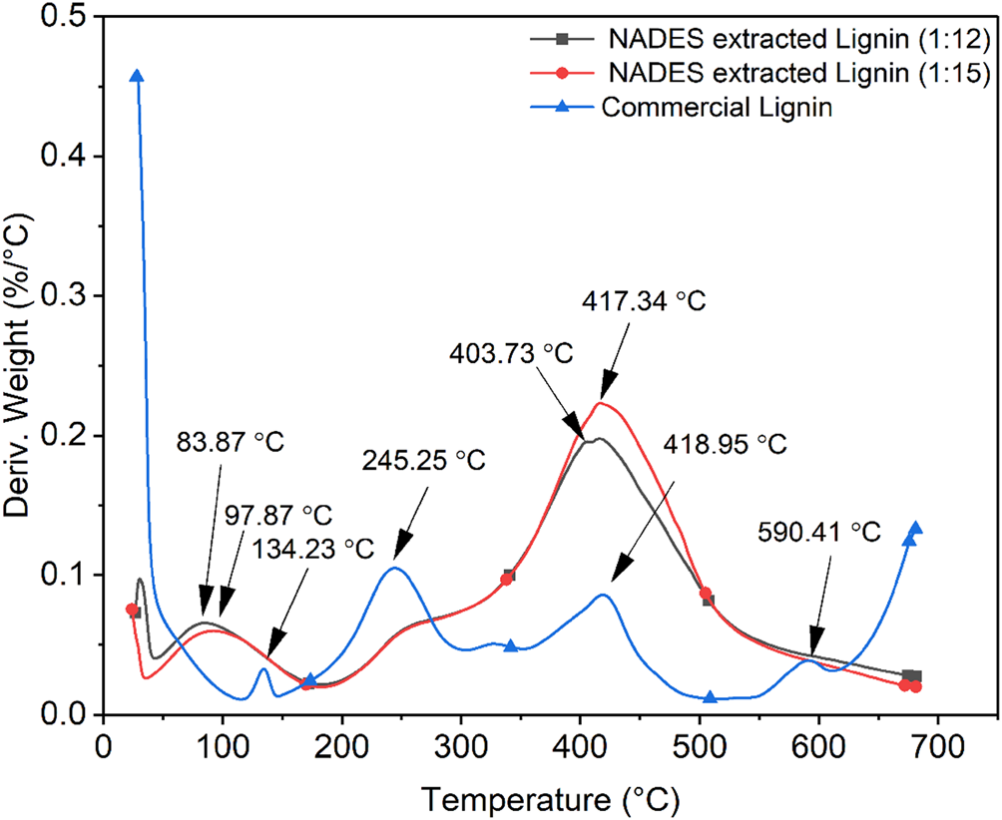
DTG curve of commercial lignin and NADES extracted lignin.

TGA and derivative thermogravimetric (DTG) curves of commercial lignin and NADES extracted lignin are represented in Figs. 6 and 7 respectively. Thermal decompositions and DTG of NADES extracted lignin (1:12 and 1:15 molar ratios) and commercial lignin occurred at temperature ranging from 60 to 600 °C are summarised in Tables 5 and 6. The TGA and DTG plots represents weight loss curves decomposition. Initial weight loss was detected in the range of 60–135 °C. This is primarily because of the moisture present which is removed when the sample is first heated up and volatile material present^40–42^. Beyond this, the decomposition temperature is observed around 200–350 °C and is associated with the destruction of carbohydrates from lignin samples, which are transformed into volatile gases such as CO, CO_2_, and CH_4_. According to Chu et al.^43^, it was reported that the β-O-4 linkage happened in the temperature range of 250 to 350 °C. NADES extracted lignin of 1:15 molar ratio has a comparatively large quantity of β-O-4 linkage compared to NADES extracted lignin of 1:12 molar ratios i.e., 332.54 and 326.01 °C respectively; consequently, the sharpness may be ascribed to the breaking of β-O-4 linkage while in commercial lignin this β-O-4 linkage was not observed in this range. While for the DTG curve, it was observed at 245 °C which only appeared in commercial lignin and the peak was absent in both 1:12 and 1:15 molar ratios of NADES extracted lignin.

**Table 5:** Thermogravimetric analysis of commercial lignin and NADES extracted lignin.

Name of samples	Initial temperature (°C)	First decomposition temp. (°C)	Second decomposition temp. (°C)	Third decomposition temp. (°C)	Weight loss (%)	Residue
Commercial lignin	127.76	207.13	388.80	566.75	22.07	66.84
NADES extracted lignin (1:12)	66.50	326.01	–	–	48.89	50.36
NADES extracted lignin (1:15)	60.15	332.54	–	–	50.139	49.54

**Table 6:** DTG analysis of commercial lignin and NADES extracted lignin.

Name of samples	First peak decomposition temp. (°C)	Second peak decomposition temp. (°C)	Third peak decomposition temp. (°C)	Fourth peak decomposition temp. (°C)
Commercial lignin	134.23	245.25	418.95	590.41
NADES extracted lignin (1:12)	83.87	–	403.73	–
NADES extracted lignin (1:15)	97.87	–	417.34	–

The second decomposition temperature observed between 350 and 388 °C and occurs due to the breakdown of linked aliphatic chains^44^, and, the last i.e., third decomposition temperature was continuous at a higher temperature at 566.75 °C of commercial lignin, while lignin degrades at a very extensive heat, but with a total weight loss of about 22.07%, because of the breakdown of an aromatic component from lignin structure^45^, and also observed in both NADES extracted lignin (1:12 and 1:15 molar ratios) whereas, second and third decomposition was not observed and weight loss (%) was higher as compared to commercial lignin. Furthermore, the residue for commercial lignin (66.84) was high as compared to the residue of NADES extracted lignin (1:12 and 1:15 molar ratios) i.e., 50.36 and 49.54 respectively. The thermal analysis on NADES extracted lignin compared to commercial lignin showed different results, could be due to NADES pretreated kenaf fibre biomass which affected the results such as heating rate and use of NADES pretreated fibre. However, DTG curve highly shows deprivation of weight loss of both NADES extracted lignin and commercial lignin i.e., above 400 °C and shows the degradation of complex lignin structure. In such stage, the disruption occurs from inter-unit connections between phenolic hydroxyl, carbonyl groups, and benzylic hydroxyl which intricates in degeneration of complex lignin structure, releasing monomeric phenols in vapour phase^46^. According to Sun et al., the breakdown or condensation process of aromatic rings in lignin structures causes a progressive shedding of lignin from 400 to 800 °C^47^. Based on above discussion, lignin is constant at higher temperatures due to more branching and the production of extremely condensed aromatic structures.

### Differential scanning calorimetry

**Figure 8:**
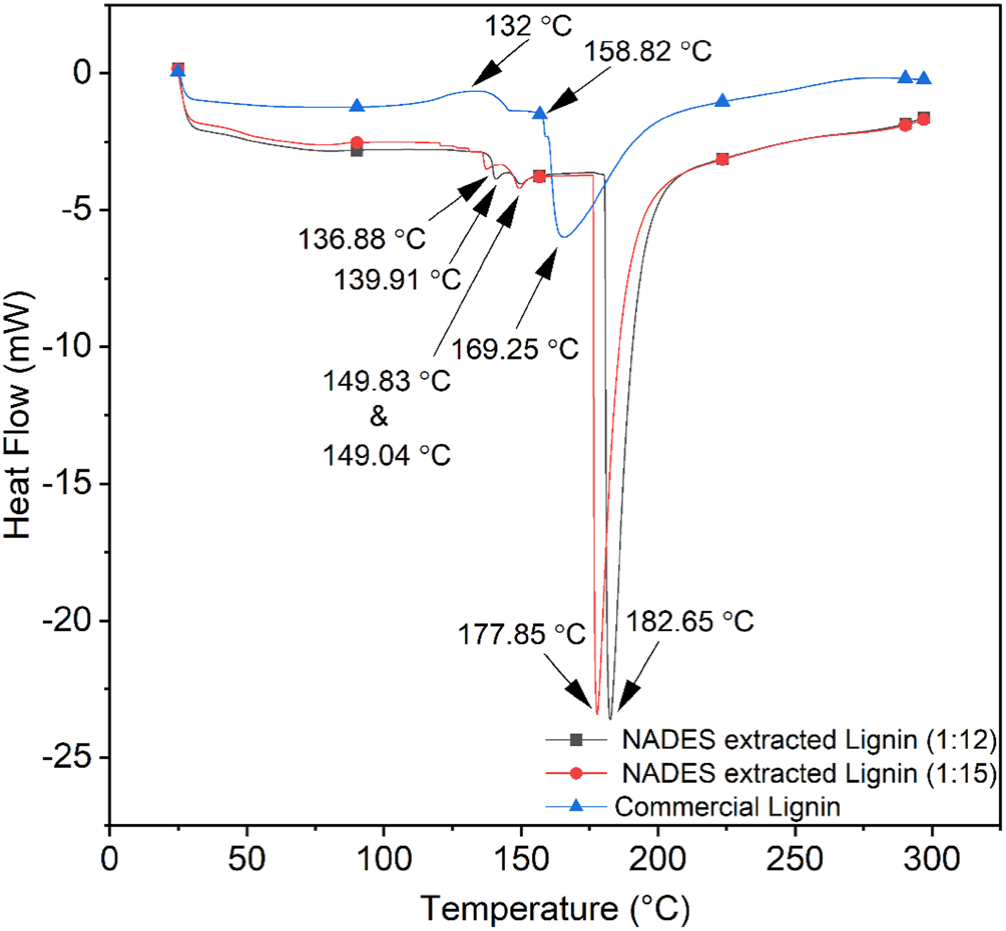
DSC thermograms of commercial lignin and NADES extracted lignin.

Differential scanning calorimetry (DSC) analysis was performed on NADES extracted lignin and commercial lignin as shown in Fig. 8 and summarised in Table 7. The temperature below 150 °C, DSC curve exhibited similar tendency, which was primarily due to moisture loss. With the increase in temperature above 150 °C, the DSC curve of commercial lignin presented a first endothermic peak at 158.82 °C which was higher than both ratios of NADES extracted lignin (1:12 and 1:15 molar ratios). The DSC curve of both ratios of NADES extracted lignin (1:12 and 1:15 molar ratios) varied with an increase in temperature, further a deep-down endothermic peak was detected above 150 °C while for commercial lignin a slight broad endothermic peak was found. Lastly, with an increase in temperature above 200 °C DSC curve was seen stable in all three samples as shown in Fig. 8.

**Table 7:** Showing DSC analysis and enthalpy of NADES extracted lignin and commercial lignin from kenaf fibre biomass.

Name of samples	Exothermic temp. (°C)	First endothermic temp. (°C )	Second endothermic Temp. (°C )	Third endothermic temp. (°C )	Enthalpy (J/g)
Commercial lignin	132.19	158.82	169.25	211.85	67.36
NADES extracted lignin (1:12)	–	139.91	149.83	182.65	129.1
NADES extracted lignin (1:15)	–	136.88	149.04	177.85	130.2

However, enthalpy measurements of the samples obtained from DSC were observed and found higher in NADES extracted (1:15) lignin which is slightly more than NADESs extracted (1:12) lignin i.e., at 130.2 and 129.1 J/g respectively, as compared to commercial lignin where a wide difference observed in enthalpy i.e., 67.36 J/g. Hence, requiring more energy to cleavage the bonds in such lignin structures (NADES extracted lignin) resulting in a highly stable product^41^.

### UV–VIS spectroscopy

**Figure 9:**
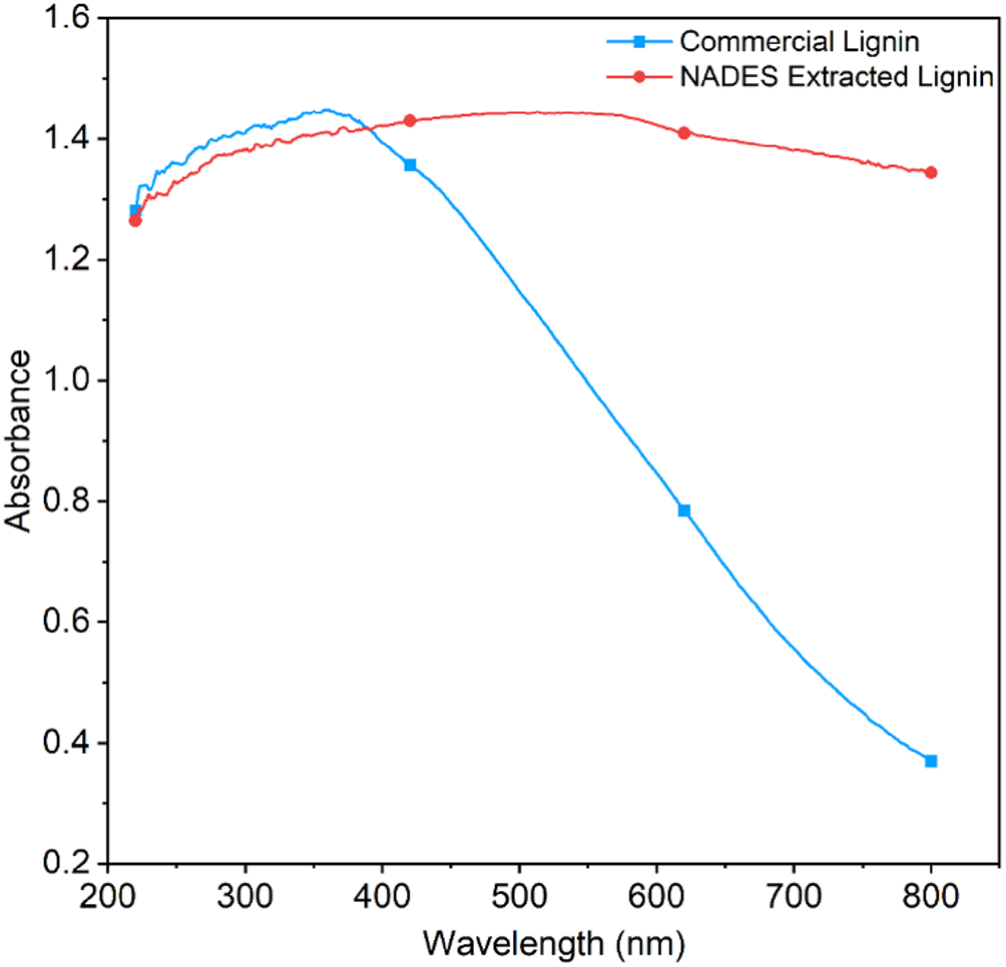
UV–Vis absorbance spectra of commercial lignin and NADES extracted lignin.

UV–Visible spectroscopy is a simple and versatile analytical technique that produces best findings in terms of structural differences of samples. Using UV–Visible spectroscopy, the spectroscopy study of lignin content extracted from NADES pretreated kenaf fibre biomass and compared with commercial lignin was examined as shown in Fig. 9.

On close examination of spectroscopy, spectra observe that the reported absorption value (i.e., 280 nm), in the ultraviolet region of lignin is shifted in both NADES extracted lignin (1:12 and 1:15 molar ratios) and commercial lignin that confirms the breaking of aromatic ring structure within the polymer. The presence of significant peaks in a study performed by Kumar et al., principal aromatic structural components of lignin i.e., coniferyl alcohol, p-coumaryl alcohol, and synapyl alcohol^48^. A quantification study was also done by Kline et al.^49^, using the ionic liquid to establish the quantity of lignin extract in wood biomass. However, once an extract of lignin was treated with 1-Butyl-3-methylimidazolium chloride, the absorption spectra were found in the visible area at 440 nm. This 1-Butyl-3-methylimidazolium chloride-initiated interference because of their high absorbance in the UV range, a multi-variation statistical technique was utilised to quantify the lignin extract. However commercial lignin shows a slightly broad peak between 300 and 400 nm that can be the presence of a carbonyl bond or ethylene-type double bond in conjunction with the benzene nucleus whereas NADES extracted lignin does not show up any peak in this range.

### X-ray diffraction analysis

**Figure 10:**
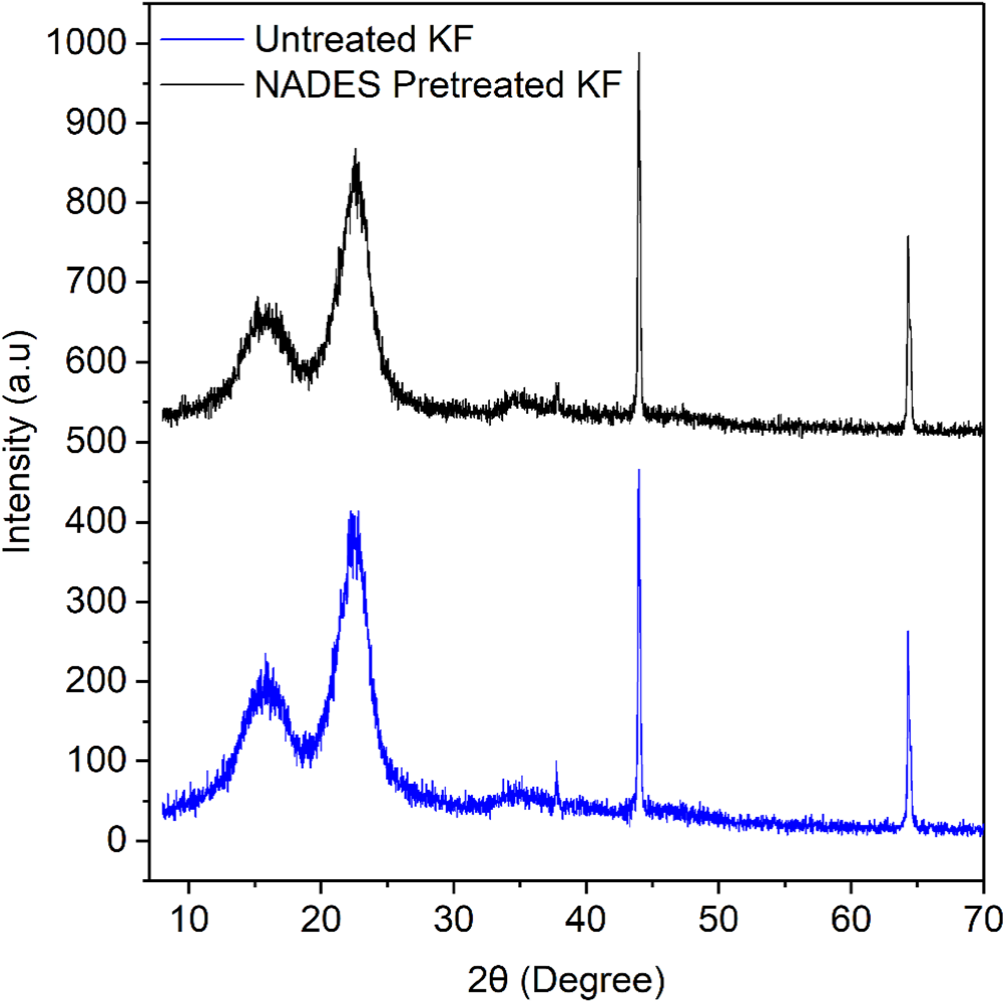
X-ray diffraction of untreated and NADES pretreated kenaf fibre biomass.

XRD spectrogram in Fig. 10, shows two peaks of untreated kenaf fibre and NADES pretreated fibre. As shown in Fig. 10, the samples shows a typical pattern with the diffraction peaks at 15.8° and 22.6°, corresponds to 110, 200 crystallographic planes respectively^50^. The CrI (%) of untreated and pretreated samples was found to be 70.33 and 69.5% respectively. According to Kumar et al.^48^, the analysis reveals that pretreatment causes a decrease in the crystallinity of cellulose, the change in crystallinity is attributed to the reduction in both amorphous and crystalline parts of cellulose, suggesting that there is partial disruption in amorphous region during pretreatment with NADESs reagent. However, in a recent study of XRD analysis, it was found that there was a rise in the crystallinity index of pretreated rice straw with mild alkali and dilute acid^51,52^.

## Conclusion

This work represents an effective and prospective method for the removal of lignin from kenaf fibre biomass utilising natural deep eutectic solvents. Lignin was extracted from NADES pretreated biomass and compared with commercial lignin. Morphological analysis showed that NADES extracted lignin exhibits smooth and wavy surfaces as compared to commercial lignin which showed irregular/uneven surfaces. The XRD results for untreated, and NADES pretreated kenaf fibre samples were determined at 70.33 and 69.5% respectively. To the best of our knowledge, this study was performed on the use of NADES as a solvent that was utilised to extract lignin from biomass, and several analyses were performed and compared with commercial lignin. NADES pretreated fibre has the potential to be a viable alternative to conventional biomass pretreatment processes. Using ecologically acceptable solvents to convert waste biomass to bioproducts necessitates a greater understanding of their effects during pretreatment. Although numerous efforts have been made to be carried out, NADES appears to be a great potential for the future generation of solvents. With greater awareness of the NADES process, applications of NADES in various fields such as biocatalysts, extraction, electrochemistry, etc., are expected to grow. Deeper understanding will be gained for future study in this field for creating customised NADES with low viscosity and good thermal stability appropriate for a variety of industrial applications. Therefore, these innovative solvents will undoubtedly have a significant impact on the development of clean, green, and sustainable products.

## Data Availability

The data that support the findings of this study are available from the corresponding author, (Jawaid, M.), upon reasonable request.
